# Bed-Sharing in the Absence of Hazardous Circumstances: Is There a Risk of Sudden Infant Death Syndrome? An Analysis from Two Case-Control Studies Conducted in the UK

**DOI:** 10.1371/journal.pone.0107799

**Published:** 2014-09-19

**Authors:** Peter S. Blair, Peter Sidebotham, Anna Pease, Peter J. Fleming

**Affiliations:** 1 School of Social & Community Medicine, University of Bristol, Bristol, United Kingdom; 2 Medical School, University of Warwick, Warwick, United Kingdom; TNO Quality of Life, Netherlands

## Abstract

**Objective:**

The risk of sudden infant death syndrome (SIDS) among infants who co-sleep in the absence of hazardous circumstances is unclear and needs to be quantified.

**Design:**

Combined individual-analysis of two population-based case-control studies of SIDS infants and controls comparable for age and time of last sleep.

**Setting:**

Parents of 400 SIDS infants and 1386 controls provided information from five English health regions between 1993–6 (population: 17.7 million) and one of these regions between 2003–6 (population:4.9 million).

**Results:**

Over a third of SIDS infants (36%) were found co-sleeping with an adult at the time of death compared to 15% of control infants after the reference sleep (multivariate OR = 3.9 [95% CI: 2.7–5.6]). The multivariable risk associated with co-sleeping on a sofa (OR = 18.3 [95% CI: 7.1–47.4]) or next to a parent who drank more than two units of alcohol (OR = 18.3 [95% CI: 7.7–43.5]) was very high and significant for infants of all ages. The risk associated with co-sleeping next to someone who smoked was significant for infants under 3 months old (OR = 8.9 [95% CI: 5.3–15.1]) but not for older infants (OR = 1.4 [95% CI: 0.7–2.8]). The multivariable risk associated with bed-sharing in the absence of these hazards was not significant overall (OR = 1.1 [95% CI: 0.6–2.0]), for infants less than 3 months old (OR = 1.6 [95% CI: 0.96–2.7]), and was in the direction of protection for older infants (OR = 0.1 [95% CI: 0.01–0.5]). Dummy use was associated with a lower risk of SIDS only among co-sleepers and prone sleeping was a higher risk only among infants sleeping alone.

**Conclusion:**

These findings support a public health strategy that underlines specific hazardous co-sleeping environments parents should avoid. Sofa-sharing is not a safe alternative to bed-sharing and bed-sharing should be avoided if parents consume alcohol, smoke or take drugs or if the infant is pre-term.

## Introduction

In the last 25 years the number of deaths due to Sudden Infant Death Syndrome (SIDS) in England & Wales has fallen from around 1600 deaths a year in 1988 to around 250 deaths a year in 2010 [1]. This 85% fall in SIDS rates has been accompanied by changes in the characteristic profile of these deaths. The proportion of deaths in families from deprived socio-economic backgrounds, among mothers who smoke during pregnancy and among pre-term infants has risen, while the peak age of death among SIDS infants found sleeping next to a parent has fallen from 3 to 2 months [2]. The *Back to Sleep* campaign, initiated in the UK in 1991, advising parents to avoid placing infants in the prone position for sleep has had a dramatic effect on the number of SIDS deaths occurring in a cot but less effect on co-sleeping deaths [2] which now account for 30–50% of all SIDS deaths [3]–[6]. Previously we have demonstrated that a proportion of these co-sleeping deaths occurred while the parent and infant slept on a sofa or chair [7] as well as a significant interaction between co-sleeping and parents recently consuming alcohol or drugs [8]. Studies have also shown an increased risk when infants slept with parents who habitually smoked [6]–[8]. However the question remains as to whether there is still a residual risk of bed-sharing in the absence of these hazardous circumstances. This question is important as it is central to public health strategy in terms of whether one should take a blanket approach to advise against bed-sharing in any circumstances as chosen for example by the American Academy of Paediatrics [9], or acknowledge that bed-sharing is a common practice and specifically target hazardous circumstances as adopted by UNICEF UK in their ‘Caring for your baby at night’ leaflet [10].

Attempts have recently been made to answer this question by amalgamating findings from previous SIDS case-control studies around the world [11] but this investigation suffered from a lack of data on hazardous co-sleeping circumstances or which parent the infant was sleeping next to. These limitations plus imputing values from whole studies where the data are missing has led to major concerns about the conclusions drawn [12]–[14]. We have previously carried out two population-based case-control studies which have collected these data in more detail; combining these data will give us more power to assess the risk associated with different circumstances of co-sleeping. The aim of this individual-level analysis, combining findings from both studies, is to quantify whether there is a risk of SIDS associated with co-sleeping in the absence of known hazards and explore the interactions with other known significant predictors of SIDS to better understand the potential risks to the infant and implications for future research.

## Methods

### Ethical approval

This study was approved by the each regional research ethics committee and by each constituent local research ethics committee for the two studies.

Both case-control studies have been fully described elsewhere [8.15] and used the same study design, similar protocols and many of the same questions and categorical responses. Both studies are population based and collected data on all sudden unexpected deaths in infancy in a defined area over a defined period and used multi-disciplinary panels to categorise the deaths as either explained or unexplained SIDS deaths (the latter being used in the analysis). The study area for the more recent investigation was one of the same English regions used in the earlier larger investigation. At this moment in time our second investigation is the most recent case-control study conducted in this field. Full ethical approval was given for both studies.

The first study was a large population control study conducted in five former English health regions (South West, Trent, Yorkshire, Wessex and Northern regions) in 1993–6 from a total study population of 17.7 million. Data were collected using a questionnaire used by research interviewers and from medical records. Bereaved families were visited within 1–2 days of the death for a narrative account and again within two weeks to complete the questionnaire. Four controls for each case were selected. The health visitor for the infant who died was asked to identify two babies on their case list born in the two weeks before the index baby and two babies born in the two weeks after the index baby. The interviewer visited each control family within a week of the index death to collect the same data as for the index case. A period of sleep (the “reference sleep”) was identified in the control infant's life in the 24 hours before the interview, corresponding to the time of day during which the index baby had died.

The second study was conducted from 2003 to 2006 in the South West Region of England with a population of 4.9 million and the cause of death was established in the same way. The control infants were weighted to be comparable with the maternal social class distribution of mothers with dependent children in Avon from the 1991 census. The age of the infants at interview and the time of day of the reference sleep were weighted to reflect approximately the ages and times of day at which infants had died.

All deaths across both studies were classified according to the Avon clinico-pathological system [16] by a multidisciplinary committee after a full paediatric necropsy to a standard protocol. Guidelines for strengthening the reporting of observational studies in epidemiology were followed [17].

### Variable definition

Co-sleeping (which includes bed- and sofa-sharing) was defined as infants sharing the same surface with at least one adult for sleep. Sofa-sharing was defined as infants sleeping on a chair or sofa (settee) with an adult. Bed-sharing was defined as infants sharing the parental bed with at least one adult. We took care to identify which adult was sleeping next to the infant; thus if the infant was sleeping between the parents we would use data on alcohol consumption or smoking from both, but if the infant was sleeping adjacent to one parent or alone with one parent then we would just use the data pertaining to that parent. The alcohol limit of no more than two units prior to the last sleep was based on recent UK recommendations of the maximum daily intake for women. Recent drug consumption was only collected in the second study so did not form part of this analysis, though has been published elsewhere [8].

The different hazards associated with co-sleeping were not mutually exclusive; some infants, for instance, slept on a sofa with an adult who smoked and had consumed lots of alcohol. These interactions have previously been explored so for this analysis a hierarchical approach of categorisation of co-sleeping was adopted based on the strength of risk reported from the two studies [8], [15] to ease interpretation of the findings. Thus sofa-sharing, quantified as the highest risk from our previous studies, was categorised regardless of whether the parents consumed alcohol or smoked, bed-sharing and alcohol consumption of more than two units was categorised regardless of smoking status and the remaining bed-sharers were categorised into smokers and non-smokers. Thus the final category representing bed-sharing in the absence of known hazards were those parents who did not co-sleep on a sofa, had not consumed more than two units of alcohol and who did not smoke. This hierarchical approach lends itself to quantifying the risks associated with three previously identified dangerous co-sleeping environments whilst also quantifying the risk of bed-sharing in the absence of these conditions.

For ease of interpretation the variables adjusted for in the multivariable analysis were dichotomised using standard definitions if available or previous definitions used in the earlier studies. These included four infant characteristics: low birthweight (<2500 g), pre-term infants (<37 weeks gestational age), gender, and whether the infant was still being breastfed when the final sleep occurred; three maternal characteristics: larger families defined as three children or more including the index infant, younger mothers aged 21 years or younger, and poor education at 16 years defined as below the General Certificate of Secondary Education level or no qualifications; and six factors pertaining to the time around the last sleep: parental report of the infant being unwell using specific signs and symptoms and scoring more than eight on the Babycheck [18], infant placed in the prone or side sleeping position, infants swaddled and use of a dummy (called a pacifier in the US). Although significant in the univariable analysis the use of pillows, tog values of infant bedding and clothes and softness of the mattress were not significant in the multivariable model, however the use of an infant or adult duvet and infants found with head or face covered by bedding were significant and have been adjusted for in the multivariable results.

An analysis of younger and older infants dichotomised around the median age allowed comparison with observations made in previous studies.

### Statistical methodology

In the first study controls were individually matched to SIDS infants for age and time of day (night time or day-time sleep) the death occurred; while for the second study, these variables were weighted rather than individually matched. Thus all univariable odds ratios quoted are adjusted for these two factors. In the multivariable analysis factors found to be significant in the previous two studies were included where available and interactions with co-sleeping investigated. The factor representing infants who slept in a different room from the parents is slightly different as it is mutually exclusive with co-sleeping and thus interacts with the reference group rather than the different co-sleeping groups. This factor was therefore added separately to the final model to observe the effect. Odds ratios (OR), 95% confidence intervals (CI) and p-values for the univariable and multivariable analysis were calculated by logistic regression using the statistical package SPSS. Models were constructed using the backward stepwise procedure for variables significant at the 5% level in the univariable analysis. Any variables with more than 5% missing data among the cases or controls were tested at the end of the modelling process.

## Results

Of the 405 SIDS infants and 1387 controls in the two studies we had data on the sleep environment in which the infant was found for 400 SIDS infants (98.8%) and 1386 controls (99.9%). Over a third of SIDS infants (36%) were found co-sleeping with an adult at the time of death compared to 15% of the controls after reference sleep. The overall risk of SIDS for infants who co-slept was more than threefold and almost fourfold when adjusted for other factors associated with SIDS (Table 1).

**Table 1:** The risk associated with co-sleeping overall and by different co-sleeping environments.

	SIDS		Controls		Univariable Risk*		Multivariable Risk†	
Overall	N	(%)	N	(%)	OR [95% CI]	P-value	OR [95% CI]	P-value
Did not co-sleep for the last sleep	255	(63.8%)	1173	(84.6%)	1.00 [Ref Group]		1.00 [Ref Group]	
Co-slept for the last sleep	145	(36.3%)	213	(15.4%)	3.19 [2.47–4.12]	<0.0001	3.91 [2.72–5.62]	<0.0001
**By different co-sleeping environments**								
Did not co-sleep for the last sleep	255	(63.8%)	1173	(84.6%)	1.00 [Ref Group]		1.00 [Ref Group]	
Co-slept on a sofa or chair	33	(8.3%)	7	(0.5%)	21.47 [9.38–49.17]	<0.0001	18.34 [7.10–47.35]	<0.0001
Bed-shared next to adult (>2 units of alcohol)	29	(7.3%)	12	(0.9%)	11.34 [5.69–22.57]	<0.0001	18.29 [7.68–43.54]	<0.0001
Bed-shared next to adult who smoked	59	(14.8%)	63	(4.5%)	4.37 [2.98–6.41]	<0.0001	4.04 [2.41–6.75]	<0.0001
Bed-shared in the absence of these hazards	24	(6.0%)	131	(9.5%)	0.86 [0.54–1.36]	0.51	1.08 [0.58–2.01]	0.82

*Adjusted for infant age and whether a day or night sleep. The logistic regression model using all 1786 individuals.

† Adjusted for infant age and whether a day or night sleep as well as infant characteristics: birthweight <2500 g, pre-term, male gender and currently breastfeeding, maternal characteristics: larger families (≥3 children), younger mothers (≤21 years) and poor maternal education (<GCSE or no qualification) factors at the time of the last sleep: infant unwell (scoring more 8 or more on the Babycheck), infant placed prone or side, infant swaddled, use of a duvet, use of a dummy and infant found with head covered.

The logistic regression model using 1700/1786 (95.4%) individuals.

When categorised by co-sleeping environment, the multivariable risk of co-sleeping with an adult on a sofa or chair, or with an adult who had consumed more than two units of alcohol was 18 times greater than those who did not co-sleep; and four times greater for those who slept next to a parent who smoked. There was no significant multivariable risk of bed-sharing in the absence of these hazards (OR = 1.1 [95% CI: 0.6–2.0]). Including a variable for infants sleeping in a separate room in the multivariable model alters the reference group and increased the risk associated with each co-sleeping environment; OR = 28.8 [95% CI: 10.9–76.1] for those who co-slept on a sofa or chair, OR = 29.7 [95% CI: 12.0–73.6] for those who slept next to someone who consumed more than two units of alcohol and OR = 6.2 [95% CI: 10.9–76.1] for those who slept next to someone who smoked. The risk associated with those who bed-shared in the absence of these hazards increased but was not statistically significant (OR = 1.6 [95% CI: 0.9–3.2], p = 0.14). None of the variables significant in the multivariable model had more than 5% missing data and over 95% of the data were used in the final model presented.

The interactions between variables used in the model and whether the infant was found co-sleeping were examined in Table 2. There was no interaction between co-sleeping and low birth weight, and although the risk for pre-term infants who co-slept was more marked (OR = 7.0 [95% CI: 3.0–16.4]) than those who did not (OR = 3.9 [95% CI: 2.6–5.8]), the interaction did not reach statistical significance (p = 0.20). Co-sleeping in general was more common among males, and interestingly any male predominance among SIDS infants was confined to those who did not co-sleep. Similarly breastfeeding was more common among those who co-slept and its protective effect was only found among those who slept alone (OR = 0.3 [95% CI: 0.2–0.5]). There was no significant interaction with maternal characteristics and co-sleeping, although larger families, younger mothers and poorer maternal education were slightly more prevalent in those who did not co-sleep. There was no significant interaction between co-sleeping and several of the factors observed at the time of the last sleep, although the prevalence of day-time sleeps and infants found with head covered by bedding was lower for both SIDS infants and controls who co-slept. The difference in infants being unwell or using a duvet was slightly more marked among those who did not co-sleep but the interaction was not significant. A significant interaction was found with dummies in that the apparent protective effect was mainly confined to those who co-slept (OR = 0.3 [95% CI: 0.2–0.5]) although was just significant for those who did not (OR = 0.8 [95%CI: 0.6–0.997]). Interestingly the risk associated with placing infants prone was absent among those who co-slept (OR = 0.4 [95% CI: 0.2–1.2]) but strongly significant among those who did not (OR = 11.3 [95% CI: 7.0–18.4]), yielding a highly significant interaction (p<0.0001). There was also a strong interaction (p<0.0001) with infant age; co-sleeping was a much greater risk for those infants younger than the median age of 98 days (OR = 3.3 [95% CI: 2.1–5.3]).

**Table 2:** Interactions with co-sleeping and significant predictors of SIDS.

	Infants who co-slept				Infants who did not co-sleep				Interaction*
	SIDS		Controls		SIDS		Controls		P-value
Infant Characteristics	N	(%)	N	(%)	N	(%)	N	(%)	
Low Birthweight <2500 g	29/145	20.0%	11/210	5.2%	58/255	22.7%	57/1168	4.9%	0.79
Pre-term (<37 weeks gestation)	29/145	20.0%	7/202	3.5%	47/253	18.6%	65/1165	5.6%	0.20
Male gender	91/145	62.8%	139/213	65.3%	163/255	63.9%	589/1173	50.2%	0.01
Still breastfeeding prior to last sleep	64/145	44.1%	98/213	46.0%	24/255	9.4%	287/1172	24.5%	0.001
**Maternal Characteristics**									
Larger families (≥3 children)	56/145	38.6%	32/213	15.0%	114/255	44.7%	272/1173	23.2%	0.33
Younger mothers (≤21 years)	41/145	28.3%	31/213	14.6%	87/255	34.1%	161/1173	13.7%	0.27
Poor maternal education†	53/135	39.3%	30/211	14.2%	101/247	40.9%	232/1169	19.8%	0.27
**Factors at the time of the last sleep**									
Whether last sleep was a day or night sleep	11/145	7.6%	12/213	5.6%	56/255	22.0%	212/1173	18.1%	0.87
Infant placed in the side position to sleep	50/141	35.5%	58/212	27.4%	92/251	36.7%	312/1170	26.7%	0.75
Infant found with head covered by bedding	11/134	8.2%	2/212	0.9%	42/246	17.1%	36/1164	3.1%	0.64
Infant swaddled for sleep	24/143	16.8%	16/213	7.5%	41/251	16.3%	108/1173	9.2%	0.52
Infant being unwell‡	41/145	28.3%	31/213	14.6%	87/255	34.1%	161/1173	13.7%	0.27
Infant covered with a duvet	72/142	50.7%	88/212	41.5%	78/253	30.8%	187/1173	15.9%	0.07
Infant used a dummy for sleep	29/134	21.6%	102/211	48.3%	106/249	42.6%	580/1172	49.5%	0.001
Infant placed in the prone position to sleep	5/141	3.5%	17/212	8.0%	53/251	21.1%	27/1170	2.3%	<0.0001
Younger infant (<98 days) at time of last sleep	111/145	76.6%	106/213	49.8%	110/255	43.1%	566/1173	48.3%	<0.0001

*P-value of the interactive term (variable of interest x co-sleeping variable) in a model including both these factors as well as infant age and, whether a day or night sleep.

† defined as qualifications below those expected at 16 years old (i.e. below GCSE level or no qualifications).

‡ defined as those infants scoring 8 or more on the Babycheck indicating the infant was unwell.

Table 3 splits the data to look at the risk of co-sleeping in younger and older infants; dichotomising by using the median of 98 days old. Overall the risk of co-sleeping among the younger infants increased to fivefold while the risk among the older infants became non-significant. Looking in more detail at the different co-sleeping environments, the numbers in some of the categories were very small so caution needs to be taken regarding the point estimates. The risk of co-sleeping with a parent on a sofa or chair was high regardless of infant age. The risk of bed-sharing next to a parent who had consumed more than two units of alcohol was higher among younger infants, but still a six-fold risk among older infants. The risk of bed-sharing next to a parent who smoked was largely confined to the younger infants while the risk of bed-sharing in the absence of these hazards was not quite significant among the younger infants (OR = 1.6 [95% CI: 0.96–2.7]) and seemingly protective among the older infants, albeit the numbers are very small. Only one SIDS death (0.6%) occurred beyond 3 months of age when bed-sharing in the absence of alcohol, smoking or sofa-sharing compared to 8.5% amongst the controls; even if we just use the upper confidence interval, the risk of SIDS halved in this particular group of infants (OR = 0.1 [95% CI: 0.01–0.5]).

**Table 3:** The risk associated with co-sleeping overall and by different co-sleeping environments in younger and older infants.

	Younger infants (<98 days)						Older infants (≥98 days)					
	SIDS		Controls		OR [95% CI]*	P-value	SIDS		Controls		OR [95% CI]*	P-value
Overall	N	(%)	N	(%)			N	(%)	N	(%)		
Did not co-sleep for the last sleep	110	49.8%	566	84.2%	1.00 [Ref]		145	81.0%	607	85.0%	1.00 [Ref]	
Co-slept for the last sleep	111	50.2%	106	15.8%	5.24 [3.71–7.39]	<0.0001	34	19.0%	107	15.0%	1.40 [0.91–2.15]	0.13
**Different co-sleeping environments**	N	(%)	N	(%)			N	(%)	N	(%)		
Did not co-sleep for the last sleep	110	49.8%	566	84.2%	1.00 [Ref]		145	81.0%	607	85.0%	1.00 [Ref]	
Co-slept on a sofa or chair	22	10.0%	5	0.7%	21.44[7.93–58.04]	<0.0001	11	6.1%	2	0.3%	23.86 [5.22–109.2]	<0.0001
Bed-shared next to adult (>2 units of alcohol)	19	8.6%	5	0.7%	19.35 [7.05–53.11]	<0.0001	10	5.6%	7	1.0%	6.38 [2.38–17.12]	0.0002
Bed-shared next to adult who smoked	47	21.3%	26	3.9%	8.93 [5.27–15.14]	<0.0001	12	6.7%	37	5.2%	1.42 [0.72–2.79]	0.32
Bed-shared in the absence of these hazards	23	10.4%	70	10.4%	1.62 [0.96–2.73]	0.07	1	0.6%	61	8.5%	0.08 [0.01–0.52]	0.009

*Adjusted for infant age and whether a day or night sleep.

## Discussion

There was no significantly increased risk for SIDS associated with bed-sharing in the absence of sofa-sharing, alcohol consumption and smoking. In infants aged less than 3 months the same proportion of SIDS infants and control infants bed-shared in the absence of these hazardous conditions and the difference was not significant. Conversely, bed-sharing in the absence of other hazards was significantly protective for infants older than 3 months; a finding that was unexpected and has not been previously reported to our knowledge. Notably, the risk associated with infants co-sleeping on a sofa or sleeping next to an adult in the parental bed who had consumed more than two units of alcohol was a magnitude higher than most risk factors associated with SIDS. Both of these environments pose a risk to the infant regardless of infant age. The reasons as to why infants are at increased risk when sleeping next to a smoker are not clear, but this risk seems to be far greater in the younger infants.

There are limitations to this secondary analysis of observational data, not least the attendant bias that such studies introduce. We have largely established potential associations rather than definitive causal factors and can only interpret the findings in terms of the factors we have recorded. The studies are also ten years apart although many of the risk factors are in the same direction and for some the magnitude has changed very little. Combining observational data from different SIDS studies can be difficult [11] but was less of a problem for this analysis as many of the questions and responses were worded exactly the same with similar study protocols and the same techniques for identifying and defining the deaths. The same comprehensive notification system was in place for both studies and the multi-disciplined panel of experts used the same classification system to clearly identify SIDS [16]. There was also a relatively small amount of missing data allowing us to conduct the analysis without the need to impute. Information on recent parental drug consumption needs to be collected when investigating co-sleeping deaths; this renders our current observations a conservative estimate of potential hazardous environments. Data on recent drug consumption, collected in our second study, suggests at least some of the risk we currently apportion to bed-sharing and smoking also involves the use of recreational drugs [8]. The combined dataset is relatively large for case-control studies and is population-based with very few missed deaths during the study period but is only large enough to look at a dichotomy of interactions amongst the multiple categories of co-sleeping deaths and even then the numbers for some categories may be small as reflected by the large confidence intervals of risk estimates.

The hazardous circumstances in which some co-sleepers were found and the interaction of significant predictors of SIDS with co-sleeping suggests we need to look closer at accidental asphyxia as a potential causal mechanism in these deaths. Our UK definition of SIDS adheres strictly to the Avon clinic-pathological classification [16] system using a multi-disciplinary panel to review every death in both studies and this analysis excludes explained deaths but it is recognised that without such thorough review some of these deaths may be classed as accidental or come under the wider umbrella of Sudden Unexpected Deaths in Infancy in other countries. Infants placed prone, male preponderance and lack of breastfeeding are common predictors of SIDS deaths and the absence of significance in these factors among those found co-sleeping may suggest a different mode of death than those SIDS infants found in a cot [19] although this could equally be related to the practice of co-sleeping itself. In a large cohort of healthy infants we have previously shown that parents are more likely to bedshare with a male infant and more likely to breastfeed [20] and this is reflected among the controls in this analysis ameliorating significance in these factors. In a longitudinal investigation of consecutive SIDS deaths over a 20 year period [2] we have also shown that co-sleeping breastfeeding mothers were less likely to place infants prone prior to the “*Back to Sleep*” campaign which partly explains the smaller reduction in these deaths than in cot-sleeping infants after the campaign. The lower proportion of SIDS infants using dummies for the last sleep was mainly restricted to those who co-slept and this novel finding was unexpected. The difference in prevalence of habitual dummy use between case and control infants is almost absent in our studies which may suggest the differences found for the last sleep is a marker of change in routine and co-sleeping in the parental bed or on a sofa might be an indicator of disruption in infant care practices that otherwise would largely go unmeasured. The interaction with young infant age suggests vulnerability, especially when we take into account the hazardous circumstances in which some of these deaths occur, thus the possibility that the causal mechanism of death in such circumstances may be different to that for solitary sleeping infants [21] needs further investigation.

An important implication of our findings is that to give blanket advice to all parents never to bed-share with their infant does not reflect the evidence. There is a danger that such advice could influence parents to seek alternative, more dangerous sleep surfaces such as a sofa. In our study in 2003–6 a number of families whose infants died informed us that they had been advised not to bed-share and thus fed the infant (and fell asleep) on a sofa. Aggressive anti-bed-sharing campaigns in both the United States and the United Kingdom depicting parental bed headboards as tombstones, mothers as meat cleavers sleeping next to the infant and parents as ogres in fairytales have been roundly condemned by the SIDS research community [22] but even a more conservative campaign can give the impression that bed-sharing is somehow innately wrong. Of course we should inform the public about risks that can be associated with bed-sharing, but bed-sharing is a widespread socio and cultural norm [23], [24]; giving across the board advice to simply not do it negates the option of highlighting the specific and highly significant risks we have found. There is also ample evidence of an interdependent positive relationship between bed-sharing and breastfeeding [20], [25] with its inherent advantages to the infant that needs to be considered in addition to the possible risk of SIDS.

## Conclusion

The evidence presented here is in line with the current public health messages promoted by the UK Lullaby Trust in their ‘Safer sleep for babies’ advice, and as outlined by UNICEF UK in their ‘Caring for your baby at night’ leaflet for parents, and their health professional guidelines [10]. In the UNICEF leaflet bed-sharing is acknowledged as something parents do either intentionally or unintentionally. The leaflet makes clear that bed-sharing is inappropriate if parents consume alcohol, take drugs or smoke, or if the infant is pre-term. The leaflet also makes clear that sofa-sharing is not a safe alternative to bed-sharing. The risk of bed-sharing and SIDS in the absence of these hazardous conditions appears to be minimal; more effort therefore needs to go into advising parents on the very real dangers associated with bed-sharing in these particular hazardous conditions.
